# Stroke burden and attributable risk factors in China, 1990–2019

**DOI:** 10.3389/fneur.2023.1193056

**Published:** 2023-05-24

**Authors:** Wenxin Tian, Guanghan Zhu, Wenbo Xiao, Bei Gao, Wenli Lu, Yuan Wang

**Affiliations:** ^1^School of Public Health, Department of Epidemiology and Health Statistics, Tianjin Medical University, Heping District, Tianjin, China; ^2^Tianjin Key Laboratory of Environment, Nutrition and Public Health, Tianjin Medical University, Heping District, Tianjin, China; ^3^Center for International Collaborative Research on Environment, Nutrition and Public Health, Tianjin Medical University, Heping District, Tianjin, China

**Keywords:** stroke, ischemic stroke, hemorrhagic stroke, risk factor, global burden of disease, China

## Abstract

**Background and purpose:**

Understanding the temporal trends of stroke burden and its attributable risk factors are essential for targeted prevention strategies. We aimed to describe the temporal trends and attributable risk factors of stroke in China.

**Methods:**

Data on the stroke burden [incidence, prevalence, mortality, and disability-adjusted life years (DALYs)] and the population-attributable fraction for stroke risk factors from 1990 to 2019 were obtained from the Global Burden of Disease Study 2019 (GBD 2019). We analyzed trends in the burden of stroke and its attributable risk factors from 1990 to 2019, and the characteristics of stroke-attributable risk factors by sex, age group, and stroke subtype.

**Results:**

From 1990 to 2019, the age-standardized incidence, mortality, and DALY rates for total stroke decreased by 9.3% (3.3, 15.5), 39.8% (28.6, 50.7), and 41.6% (30.7, 50.9) respectively. The corresponding indicators all decreased for intracerebral hemorrhage and subarachnoid hemorrhage. The age-standardized incidence rate of ischemic stroke increased by 39.5% (33.5 to 46.2) for male patients and by 31.4% (24.7 to 37.7) for female patients, and the age-standardized mortality and DALY rates remained almost unchanged. The three leading stroke risk factors were high systolic blood pressure, ambient particulate matter pollution, and smoking. High systolic blood pressure has remained the leading risk factor since 1990. The attributable risk of ambient particulate matter pollution shows a clear upward trend. Smoking and alcohol consumption were important risk factors for men.

**Conclusion:**

This study reinforced the findings of an increased stroke burden in China. Precise stroke prevention strategies are needed to reduce the disease burden of stroke.

## 1. Introduction

As a country with one-fifth of the world's population, China has the highest global stroke burden and has undergone rapid epidemiological changes in recent decades (1, 2). In China, although the age-standardized incidence and mortality rates for total stroke decreased from 1990 to 2019, the corresponding crude rates increased sharply over the same period, which led to dramatic growth in stroke prevalence and a substantial burden for patients and the healthcare system (3). With added challenges of changing lifestyles and population aging, the overall stroke burden in China is projected to increase without effective prevention strategies (1). Therefore, timely updated disease burden results are critical for evidence-based treatment planning and resource allocation (4).

This study aimed to analyze the incidence, prevalence, deaths, and DALYs of total stroke and its subtypes, including ischemic stroke (IS), intracerebral hemorrhage (ICH), and subarachnoid hemorrhage (SAH) in China (mainland) based on the GBD study 2019. We analyzed the absolute numbers, rates, and age-standardized rates from 1990 to 2019 and compared them across different groups based on age and sex. In addition, we provided a detailed insight into the risk factors contributing to stroke burden from 1990 to 2019 and compared the population-attributable fractions (PAFs) in different cohorts grouped by age and sex.

## 2. Methods

### 2.1. Data resource

Data on stroke burden in China were obtained from the GBD Results Tool (https://vizhub.healthdata.org/gbd-results). The GBD Study Group used multiple data sources and complex statistical models to mitigate the effects of low availability and poor-quality data. The general methodology used in the GBD study has been described in detail in previously published studies (5, 6). In this study, we used GBD 2019 data to estimate the temporal trend of stroke burden and the PAFs of stroke-related risk factors. In the stroke burden analysis, we used four standard epidemiological measures, namely, incidence, prevalence, mortality, and disability-adjusted life years (DALYs). In the stroke-related risk factors analysis, we examined the PAFs of stroke risk factors by stroke subtype and the top 10 risk factors for IS, ICH, and SAH by groups based on age and sex.

### 2.2. Stroke definition

In this study, stroke was defined according to the World Health Organization's definition and was modeled into three subcategories: ischemic stroke, intracerebral hemorrhage, and subarachnoid hemorrhage (7). Ischemic stroke is defined as any first-ever vascular event that results in reduced blood flow to brain tissue and leads to infarction, thromboembolic stroke, or atherosclerotic stroke. Hemorrhagic strokes (ICH and SAH) are defined as non-traumatic subarachnoid or intracerebral hemorrhage (8).

### 2.3. Risk factor estimation

The PAF is the proportion of the outcome that would be removed if a risk factor had been reduced to the theoretical minimum risk exposure level (TMREL). The GBD 2019 used the comparative risk assessment (CRA) framework to estimate disability-adjusted life years (DALYs) for risk factors. The specific estimation methods are described in detail elsewhere (6, 9).

### 2.4. Statistical indicators and graphs

The age-standardized rates for incidence, mortality, prevalence, and DALYs were calculated with a global age structure in 2019. The 95% uncertainty intervals (UIs) were calculated by taking 1,000 samples from the posterior distribution of the respective step in the modeling process and reported as the 2.5th and 97.5th values for each estimate. This study generated all figures through GraphPad Prism 8.0, Origin 2022, and Excel 2021.

## 3. Results

### 3.1. Temporal trends of stroke burden according to sex, stroke types, and age groups in China

**Figure 1:**
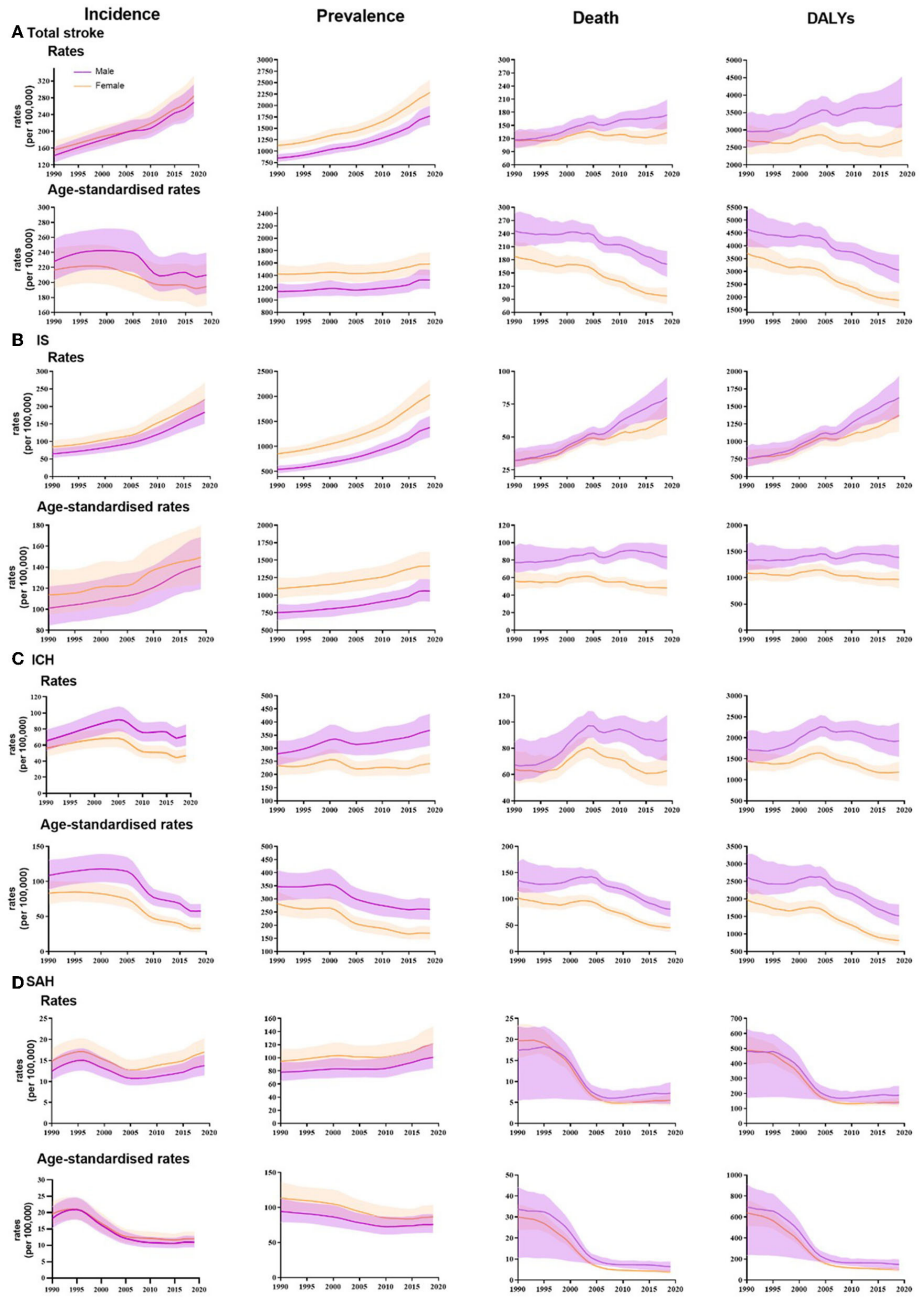
Temporal patterns of incidence, prevalence, deaths and DALYs rates and age-standardized rates of total stroke and its subtypes in China, by sex, 1990-2019 **(A)** Total stroke. **(B)** IS: ischemic stroke. **(C)** ICH: intracerebral hemorrhage. **(D)** SAH: subarachnoid hemorrhage.

**Figure 2:**
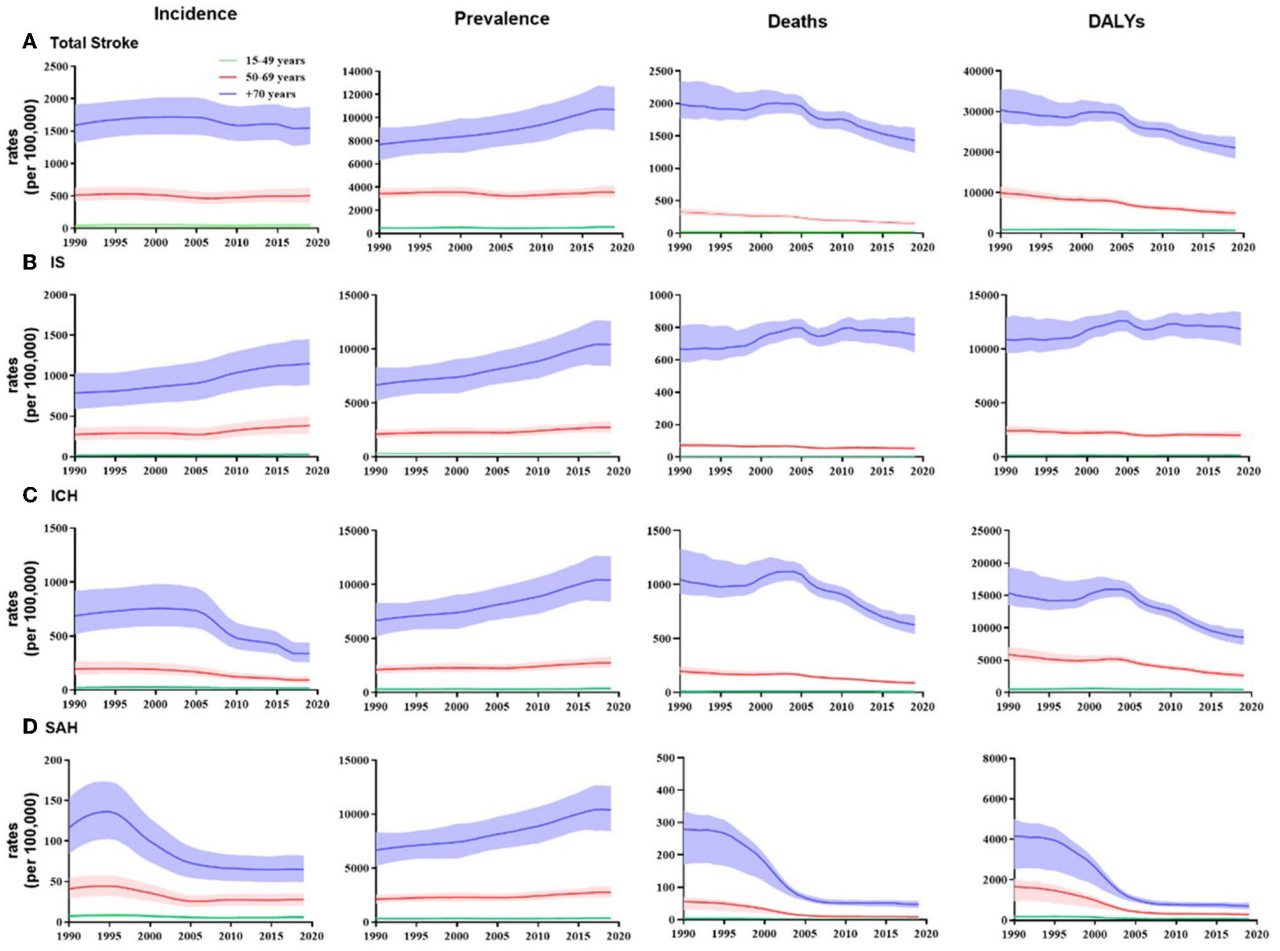
Temporal patterns of incidence, prevalence, deaths, and DALY rates of total stroke and its subtypes in China, 1990–2019, by three age groups (15- to 49-year age group, 50- to 69-year age group, and 70 years or older group). **(A)** Total stroke; **(B)** IS, ischemic stroke; **(C)** ICH, intracerebral hemorrhage; **(D)** SAH, subarachnoid hemorrhage.

In 2019, there were 3.94 (95% UI 3.43 to 4.58) million new stroke cases in China. The crude incidence rate of total stroke was higher in female patients than male patients, but the age-standardized incidence rate was higher in male patients (Supplementary Table 1). In stroke subtype analysis, the age-standardized incidence rates were higher for both ICH and SAH in men than in women, with larger differences for ICH [57.7 per 100,000 (48.6 to 67.7) for men and 33.1 per 100,000 (27.6 to 39.5) for women]; for IS, conversely, the age-standardized incidence rates were higher in women [141.1 per 100,000 (118.9 to 168.6) for men and 149.4 per 100,000 (124.7 to 179.8) for women] (Figure 1). From 1990 to 2019, the age-standardized incidence rate of total stroke decreased by 9.3% (3.3 to 15.5). Significant increases were found in the age-standardized incidence rates of IS for both men [39.5% (33.5 to 46.2)] and women [31.4% (24.7 to 37.7)] (Figure 1). In age-specific analysis, in 2019, the subgroup aged 80–84 years [1,326.4 (944.9 to 1,801.7) per 100,000 population] had the highest IS incidence. The incidence rate for ICH and SAH increased with age and finally reached 541.4 (389.7 to 757.8) per 100,000 population and 101.3 (77.5 to 130.6) per 100,000 population, respectively, in the subgroup aged 85 years or older (Supplementary Figure 1). From 1990 to 2019, the 50–69 age group and the 70 or older age group showed a consistent upward trend for IS incidence (Figure 2).

There were 2.19 (1.89 to 2.51) million deaths attributable to total stroke in 2019: 1.26 million (1.04 to 1.51) deaths in men and 0.93 (0.75 to 1.12) million deaths in women (Supplementary Table 1). Age-standardized mortality rates were higher in men than in women for total stroke and subtypes. From 1990 to 2019, the age-standardized mortality rate of total stroke decreased by 39.8% (28.6 to 50.7), with the largest decrease in women. The age-standardized mortality rates of ICH and SAH all decreased since 1990, while the age-standardized mortality rates of IS had not changed significantly (Figure 1). Across all stroke subtypes, mortality rates tended to increase with age (Supplementary Figure 1).

There were 28.76 (25.60 to 32.21) million prevalent cases in 2019 in China. The age-standardized prevalence of total stroke was higher in women than in men (Supplementary Table 1). The age-standardized prevalence of IS and SAH was higher in women, while for ICH it was higher in men. From 1990 to 2019, the age-standardized prevalence of IS increased by 40.6% (30.8 to 51.1) for men and 29.8% (21.4 to 39.5) for women (Figure 1). In the age-specific analysis, the highest prevalence for IS was found in the subgroup aged 80–84 years (13116.8, 95% UI 10496.0 to 16168.4), while for ICH, the highest prevalence was found in the 60- to 64- and 65- to 69-years age group (840.2 [660.2 to 1068.6]; 842.74 [655.9 to 1063.8] per 100,000 population, respectively). The SAH prevalence was relatively the same in the subgroup aged 65–69 years or older (Supplementary Figure 1). For all disease subtypes, there was a clear upward trend in prevalence in the 70 years old or above age group (Figure 2)

In 2019, the DALYs of total strokes were 45.95 (39.81 to 52.34) million in China. Women had fewer stroke DALYs than men. The DALY rate of total stroke for women was lower than that for men, and this gap was more obvious after age standardization. From 1990 to 2019, the age-standardized DALY rates for stroke decreased for both men and women, with the largest change occurring in women [−49.4% (−59.6 to −36.3)]. In the subtype analysis, the age-standardized DALY rates trended downward for both ICH and SAH. Still, there was no significant change for IS (Figure 1). The DALY rates of IS, ICH, and SAH all increased with age and reached the highest rates in the subgroup aged 85 years or older (Supplementary Figure 2).

### 3.2. Risk factors of strokes

**Figure 3:**
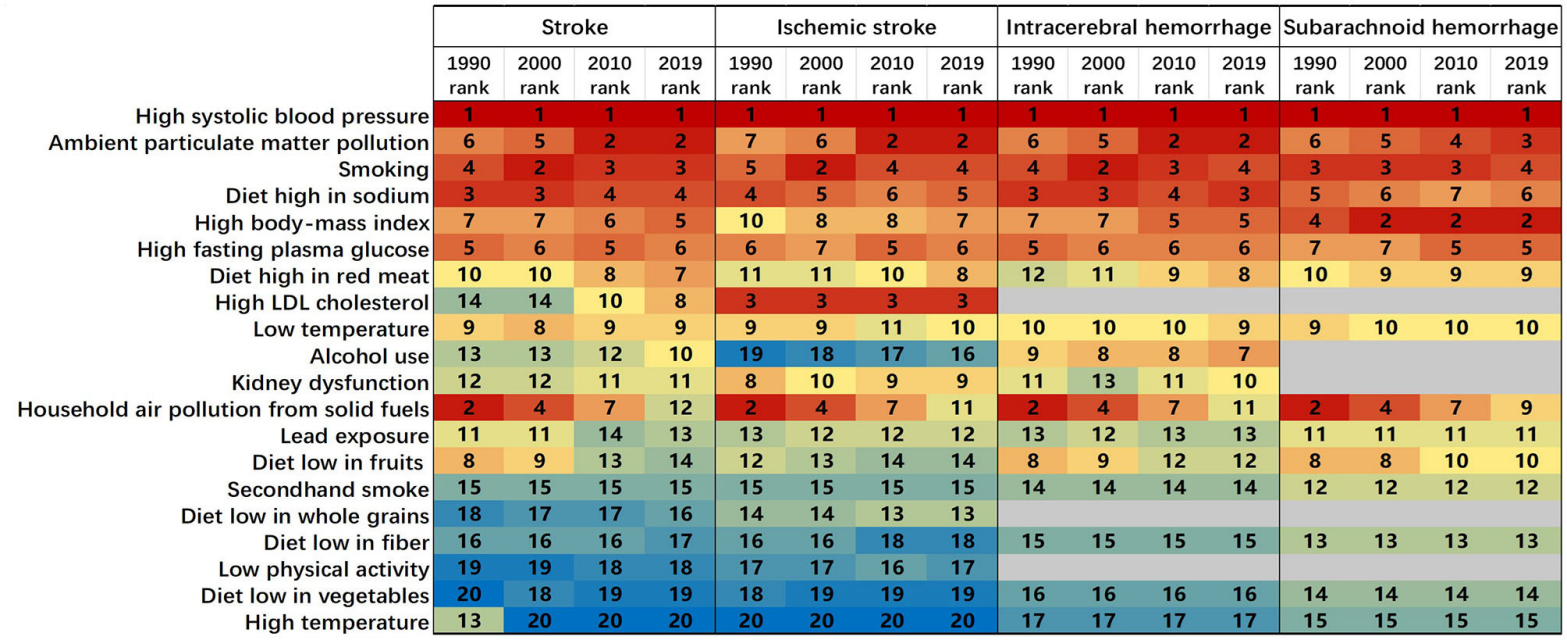
Age-standardized stroke-related DALYs attributable to 20 risk factors in China, for both sexes, in 1990, 2000, 2010, and 2019 separately. Numbers show the ranking level (1 = highest and 20 = lowest) by the number of DALYs attributable to the corresponding risk factors. High LDL cholesterol, a diet low in whole grains, and low physical activity were not assessed for intracerebral hemorrhage. High LDL cholesterol, alcohol use, kidney dysfunction, a diet low in whole grains, and low physical activity were not considered for subarachnoid hemorrhage. DALY=disability-adjusted life year.

As can be seen in Supplementary Figure 2, in 2019, 88.3% (95% UI 85.4 to 90.9) of total stroke DALYs were attributable to 20 risk factors in China. The PAF for total stroke DALYs attributable to all risk factors was similar for IS [87.0% (82.9 to 91.2)], ICH [89.6% (86.3 to 92.3)], and SAH [87.1% (83.7 to 90.0)]. Figure 3 illustrates the ranking of PAFs for stroke risk factors. From 1990 to 2019, high systolic blood pressure remained the leading risk factor for total stroke and its subtypes. There was an increase in PAF of stroke-related DALYs due to ambient particulate matter pollution (in all stroke subtypes), high body-mass index (BMI), a diet high in red meat, high LDL cholesterol (increased in total stroke, remained unchanged in IS), and alcohol use (both increased in IS and ICH). High LDL cholesterol remained the third rank for IS from 1990 to 2019. Alcohol use was mainly associated with ICH.

**Figure 4:**
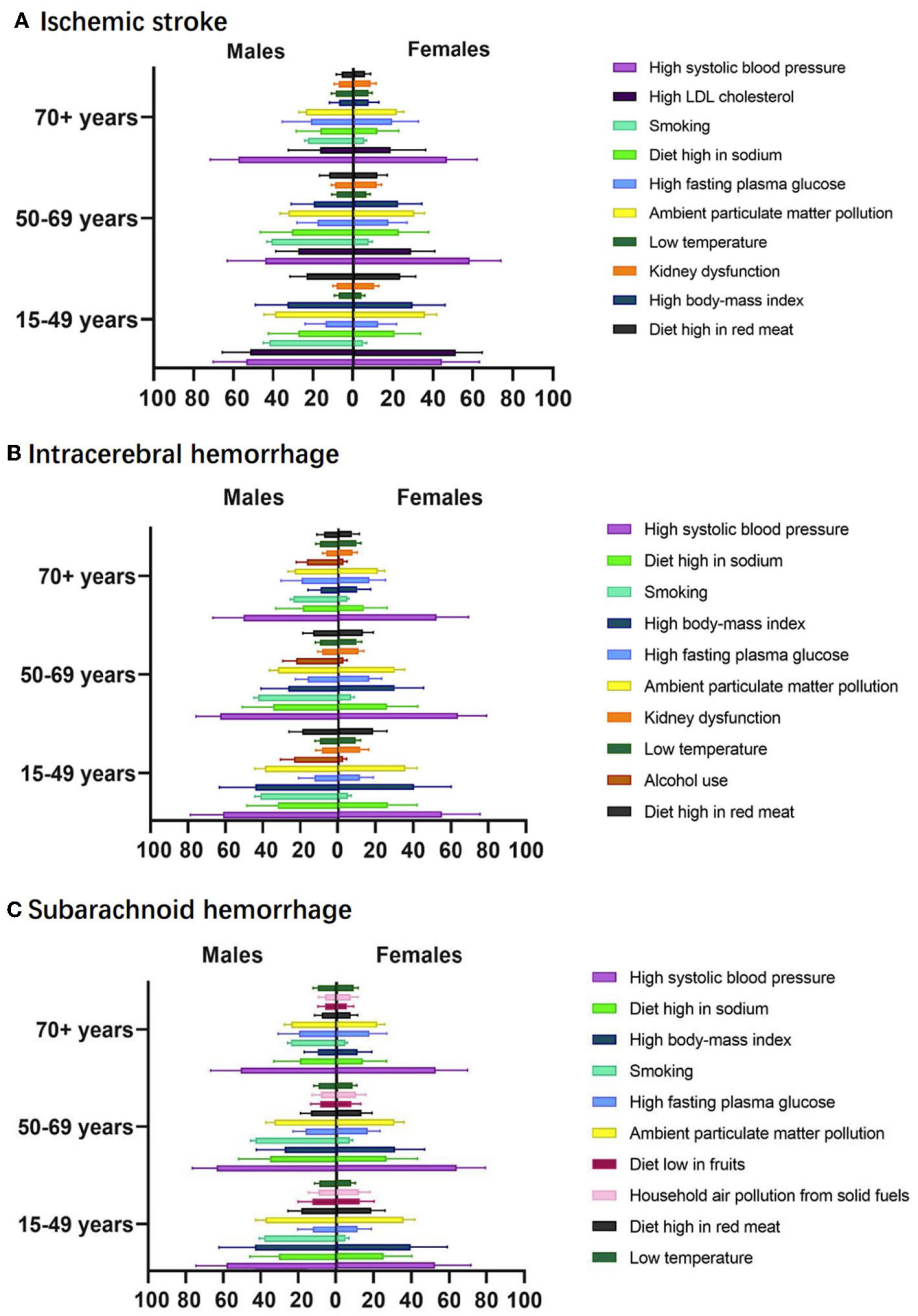
The proportion of DALY attributable to leading specific risk factors by sexes and age groups, 2019, for ischemic stroke **(A)**, intracerebral hemorrhage **(B)**, and subarachnoid hemorrhage **(C)**.

The proportion of DALYs attributable to the 10 leading specific risk factors of IS, ICH, and SAH in 2019 was analyzed by age and sex, as shown in Figure 4. PAF values for stroke risk factors showed certain variations within age groups. High systolic blood pressure and ambient particulate matter pollution were the leading risk factors for IS, SAH, and ICH among the three age groups. PAFs for high LDL cholesterol in IS and high body-mass index in ICH and SAH were significantly higher in the 15–49 age group. High LDL cholesterol surpassed high systolic blood pressure as the first risk factor for PAF values in women aged 15–49 years. PAFs for high systolic blood pressure were the highest in the 50–69 years age group. Concerning gender disparities, alcohol and smoking were more critical risk factors in men. PAFs for smoking were higher for men in the 15–49 [41.9 (38.8 to 45.1)] and 50–69 age groups [40.9 (38.5 to 43.3)] than 70 years or above age group [22.7 (21.1 to 24.4)].

## 4. Discussion

This study reported the burden of stroke and its attributable risk factors in China by stroke subtype, age group, and sex using GBD 2019 data. From 1990 to 2019, the age-standardized incidence, mortality, and DALY rates of stroke in China all showed decreasing trends, but crude rates of the corresponding indicators all increased over the same period. The prevalence of stroke showed increasing trends both before and after age standardization. In the stroke subtype analysis, the age-standardized incidence of IS increased continuously from 1990 to 2019, with a higher incidence in women. Age-standardized mortality and DALY rates for IS remained relatively stable over the same period. In the risk factor analysis, high systolic blood pressure remained the leading risk factor for stroke since 1990. Environmental particulate matter pollution showed a clear upward trend and became the second most important risk factor in 2019. For the group aged 15–49 years, high LDL cholesterol (in IS) and high body-mass index (in ICH and SAH) should be noted, and in the group aged 50–69 years, the highest PAF was found for hypertension. PAFs for risk factors were generally lower in the group aged 70 years and older. Regarding sex-specific differences, alcohol consumption and smoking are more important risk factors for men than for women. Smoking has high PAF values for all age groups in men.

From 1990 to 2019, the age-standardized incidence, mortality, and DALY rates for total stroke in China all trended downwards, while the corresponding crude rates continued to rise over the same period. This paradox indicates that China has made some progress in stroke prevention and treatment, but these improvements have not yet fully reversed the increasing trend in stroke burden, with the aging population contributing to this increasing trend. China's seventh census showed that 190 million people, or 13.5% of the population, were aged 65 years and over. The continuing growing aging process continues to increase the stroke burden in China. Relevant prevention and treatment measurements were conducted in China. In 2007, China set out to improve the quality of stroke care by launching the China National Stroke Registry (CNSR) registry, establishing a large prospective cohort, analyzing risk factors and post-operative recovery for each stroke subtype, and developing an early warning prediction model for stroke recurrence (10). These are effective ways to reduce the growing burden of stroke due to aging (11).

Analysis of stroke prevalence trends found that the prevalence of total stroke in China from 1990 to 2019 showed a significant upward trend both before and after age standardization. As both age-standardized morbidity and mortality rates have decreased over the same period, it is assumed that the increase in age-standardized prevalence is partly due to improvements in stroke treatment. This phenomenon suggests that prevention of post-stroke recurrence is now one of the key action objectives in stroke prevention and control. China has progressively implemented community stroke screening and health education, promoted the establishment of telestroke programs and citywide stroke/thrombolytic maps, improved health insurance coverage, increased stroke detection and access to stroke care for residents, and achieved effective results (10, 12, 13). Optimizing secondary stroke prevention, stroke rehabilitation, and personalized care is now a key direction for alleviating the burden of disease in China (14). Given the challenges posed by an aging society to stroke prevention and care, multidimensional stroke prevention and management strategies should be considered to improve the quality of health of the population through comprehensive stroke prevention and control measures in multiple dimensions, including stroke etiology, health education, control of risk factors, access to stroke care, quality of stroke care, and stroke recurrence control.

Analysis by disease subtype reveals a continuously increasing burden of IS for both men and women. The age-standardized incidence of IS increased continuously since 1990, and the age-standardized mortality and DALY rates of IS remained approximately at their initial levels in 1990. This trend is consistent with the results of a cohort study conducted in China from 1984 to 2004, which suggested that effective prevention strategies aimed at reducing the IS burden had not been successfully implemented (15). Although several studies showed the decreasing trend for age-standardized incidence and mortality rates for total stroke in China (10, 16, 17) the continuously growing trend for IS burden should be taken seriously. China should take active actions to implement effective preventive measures to reduce the burden of IS.

In gender-specific analysis, from 1990 to 2019, the age-standardized death and DALY rates were all higher for men than women among stroke subtypes. These findings suggest that men are more likely than women to experience disability and death from stroke. In stroke subtype analysis, the age-standardized incidence and prevalence of IS were all higher in women than in men. Liberale et al. (18) pointed out that there is an age- and gender-dependent distribution of the IS burden, with the incidence of IS becoming more pronounced in women over the age of 65 years. Female patients tend to have more severe outcomes and more comorbidities, resulting in lower quality of survival (19, 20). Therefore, China should pay more attention to stroke prevention and post-morbid care for women, especially female IS patients.

Recent studies have suggested a trend toward a younger onset of stroke (21). As the average age of stroke onset declines, the burden of stroke in younger generations needs to be taken seriously (21). Approximately 10–15% of IS occurred in people who were less than 50 years old, and these strokes are often referred to as “early onset stroke” or “young onset stroke” (22, 23). Ning et al. (24) assessed the trends in the incidence of first-ever stroke among adults aged 35 to 64 years in Tianjin, China. They found that adults aged 55 to 64 years had the largest percentage increase. The increasing trend in stroke incidence, especially in IS, among young adults has highlighted the need to study and understand the risk profiles in the younger generation (25, 26). This study found that from 1990 to 2019, for both total stroke and subtypes, the increasing trend in stroke incidence was most pronounced in the 70+ age group, with an increasing trend in the 50–69 age group for IS and a relatively moderate trend in all 15–49 age groups. Further studies are needed to examine whether there is a trend toward early onset stroke in China.

From 1990 to 2019, high systolic blood pressure remained the leading risk factor for stroke and its subtypes in China. Several population-based cross-sectional studies and cohort studies indicated that hypertension was the most prevalent stroke risk factor and the largest contributor to the stroke burden in China (27–29). In this study, PAF values for high systolic blood pressure were all higher for ICH and SAH than for IS in each age group. This phenomenon suggests that controlling systolic blood pressure has a more pronounced effect on controlling the disease burden of ICH and SAH, compared with that of IS (15). Therefore, prevention strategies for IS should target multiple risk factors to achieve more visible results. Recently, the definition of hypertension was revised in the Chinese Clinical Practice Guidelines for Hypertension. The diagnostic threshold for hypertension in adults was lowered to 130/80 mmHg. According to the 2018 survey data, the mean age-standardized systolic blood pressure of Chinese men was already above the threshold for hypertension and that of women was close to the threshold (132.5 mm Hg and 129.4 mm Hg, respectively) (30). One study found that either 140/90 mm Hg or ≥130/80 mm Hg increased the risk of adverse cardiovascular events (31). Therefore, it is likely that the actual burden of stroke attributable to high systolic blood pressure is higher than the current findings suggest, and studies on the association of high systolic blood pressure with stroke morbidity and mortality, and related preventive management measures need to be updated.

The PAFs for ambient particulate matter pollution increased significantly and became the second risk factor for all stroke subtypes in 2019. Particulate matter (PM) was associated with stroke incidence, mortality, and recurrence, with both short-term and long-term exposure effects. When PM exposure was compared with stroke admissions, short-term exposure to higher concentrations of PM 2.5 and PM10 significantly increased the risk of ischemic stroke, while no association was found with admissions for hemorrhagic stroke (32). A prospective cohort study in China found that chronic exposure to high levels of PM 2.5 significantly increased the risk of IS, ICH, and SAH, with a higher hazard ratio for ischemic stroke (33). PM 2.5 exposure and stroke incidence showed an approximately linear relationship (33). The long-term effects of PM exposure on stroke (1 year) were more pronounced than the short-term effects (7 days before the event of interest), with high PM exposure significantly increasing in-hospital mortality from stroke and rehospitalization of stroke patients (34, 35). Given the significant increase in PAF values for ambient particulate matter pollution, increased attention should be paid to air quality control, and long-term monitoring and control of air pollution exposure should be maintained to effectively reduce the burden of stroke-related disease.

This study found that the PAF for smoking was higher in men than in women in China, which was in line with an analysis from the National Stroke Screening Survey (36). Smoking was an important risk factor for all stroke subtypes. Among the three age groups, the highest PAF for smoking was found in men aged 50–69 years. There was a significant dose–response relationship between smoking and stroke risk (37). A community-based cross-sectional survey found that the multi-adjusted ORs for stroke all increased with the amount and duration of smoking (people aged ≥40 years), and smoking cessation appeared to substantially reduce this excess risk (38). Exposure to environmental tobacco smoke is also correlated with stroke, as husbands' smoking would increase the prevalence of stroke in non-smoker wives (38). There are an estimated 282 million smokers in China (271 million men and 11 million women) (39). The prevalence of smoking in China is currently approximately 25.1%. It was significantly higher in men than in women (47.6% vs. 1.9%) (40). Tobacco dependence, the main barrier to quitting, now accounts for 50% of the smoking population in China (40). A nationwide risk factor survey found that there was no significant change in the prevalence of smoking among men with chronic diseases between 2007 and 2018 (39). There is an urgent need for action in China to control the current high prevalence of smoking, and effective tobacco control measures are needed. Tobacco control is not only effective in controlling the incidence of stroke but also in increasing the life expectancy of the population, making it an effective measure to cope with an aging society (41). China should further strengthen the role of primary healthcare providers (HCP) in smoking cessation activities and integrate smoking cessation interventions into clinical practice to effectively control smoking rates in China and to reduce the disease burden.

In the age-specific analysis, people in the 15–49 and 50–69 age groups had higher PAFs in the behavioral and environmental risk cluster compared with PAFs in the 70 and over age group. Stroke risk factors were found to correlate with patients' ages. A stronger association was found in those younger than 75 years old, whereas in those older than 75 years, first stroke onset was mainly associated with a history of high systolic blood pressure (42). Although the increased stroke burden in the younger population accounted for only a small part of the overall increase in the whole population, younger patients may bear a greater lifetime burden of disability (due to their longer life expectancy) and have a much higher percentage of PAR for modifiable risk factors (43). Therefore, appropriate preventive measures should be taken for different age groups. For the elderly, the focus should be on blood pressure control, whereas for young adults, population-based preventive measures should be implemented, targeting blood pressure reduction, and behavioral, lifestyle, and dietary changes (43).

There were some limitations to this study. First, only first strokes were included in this study and recurrent strokes should also be considered in future burden of disease studies. Second, the data for this article are derived from the GBD study and the results can be further compared with real-world datasets. Third, there are geographical differences in the burden of stroke in China, and attention should be paid to geographical and rural-urban differences in China, with the results guiding more targeted disease prevention and control strategies.

Our results support the conclusion of an increased stroke burden in China. The stroke burden in China is characterized by age, sex, and disease subtypes. From 1990 to 2019, the stroke burden was concentrated in the 70+ age group; stroke mortality and DALY rates were higher in men, and stroke prevalence was higher in women; for stroke disease subtypes, the IS disease burden continues to increase and has not been effectively controlled. The three leading risk factors for stroke were high systolic blood pressure, ambient particulate matter pollution, and smoking. PAF values for stroke risk factors showed age- and sex-specific differences. Stroke prevention measures should be targeted at different population groups.

## Data availability statement

Publicly available datasets were analyzed in this study. This data can be found here: Institute for Health Metrics and Evaluation (IHME), Global Health Data Exchange (GHDx), Global Burden of Disease 2019 (GBD 2019) study, https://vizhub.healthdata.org/gbd-results/.

## Ethics statement

The studies involving human participants were reviewed and approved by the Tianjin Medical University. Written informed consent for participation was not required for this study in accordance with the national legislation and the institutional requirements.

## Author contributions

WT and YW contributed to the conception of the study. WX organized the database. WT, GZ, and BG performed the data visualization. WT wrote the first draft of the article. GZ, WX, BG, WL, and YW wrote sections of the article. All authors contributed to the article revision and read and approved the submitted version.
