# Supplementary material for: Stroke burden and attributable risk factors in China, 1990–2019
Source: Front Neurol. 2023 May 24;14:1193056. doi: 10.3389/fneur.2023.1193056 (PMC10245554; doi:10.3389/fneur.2023.1193056)
Supplement: Supplementary file 1 [file Data_Sheet_1.docx]

Supplementary Material

Stroke Burden and Attributable Risk Factors in China, 1990-2019

Wenxin Tian, Guanghan Zhu, Wenbo Xiao, Bei Gao, Wenli Lu, Yuan Wang ^*^

*** Correspondence:** Yuan Wang: gw_928@163.com

# Supplementary Tables

Supplementary Table 1: Number, rate, and age-standardised rate for stroke incidence, prevalence, deaths and DALYs in 2019 and percentage changes from 1990 and by sex in China

|  |  | Incidence (95%UI) | |  | Deaths(95%UI) | |  | Prevalence(95%UI) | |  | DALYs(95%UI) | |
| --- | --- | --- | --- | --- | --- | --- | --- | --- | --- | --- | --- | --- |
|  |  | 2019 | Percentage change 1990-2019 (%) |  | 2019 | Percentage change 1990-2019 (%) |  | 2019 | Percentage change 1990-2019 (%) |  | 2019 | Percentage change 1990-2019 (%) |
| Total stroke |  |  |  |  |  |  |  |  |  |  |  |  |
| Absolute numbers,  in thousands | Male | 1951  (1714, 2261) | 124.8  (109.8, 141.6) |  | 1261  (1035, 1509) | 76.9  (36.2, 129.1) |  | 12834  (11430, 14430) | 148.0  (131.9, 165.3) |  | 27103  (22271, 32864) | 49.7  (15.5, 94.5) |
|  | Female | 1984  (1719, 2323) | 122.3  (106.3, 137.8) |  | 928  (747, 1117) | 39.7  (7.0, 81.2) |  | 15926  (13997, 17869) | 147.2  (131.5, 164.1) |  | 18853  (15685, 22250) | 21.5  (-3.2, 52.1) |

Continued

|  |  | Incidence (95%UI) | |  | Deaths(95%UI) | |  | Prevalence(95%UI) | |  | DALYs(95%UI) | |
| --- | --- | --- | --- | --- | --- | --- | --- | --- | --- | --- | --- | --- |
|  |  | 2019 | Percentage change 1990-2019 (%) |  | 2019 | Percentage change 1990-2019 (%) |  | 2019 | Percentage change 1990-2019 (%) |  | 2019 | Percentage change 1990-2019 (%) |
|  | Total | 3935  (3432, 4580) | 123.5  (108.2, 139.1) |  | 2190  (1886, 2514) | 59.0  (30.5, 91.1) |  | 28760  (25600, 32213) | 147.5  (132.8, 162.9) |  | 45949  (39814, 52336) | 36.7  (13.8, 63.6) |
| Rate,  per 100 000 | Male | 269.2  (236.4, 311.9) | 89.2  (76.6, 103.4) |  | 174.0  (142.8, 208.2) | 48.9  (14.7, 92.9) |  | 1770.7  (1577.0, 1990.9) | 108.7  (95.2, 123.4) |  | 3739.3  (3072.6, 4534.1) | 26.0  (-2.7, 63.8) |
|  | Female | 284.5  (246.5, 333.0) | 82.8  (69.6, 95.5) |  | 133.1  (107.1, 160.2) | 14.9  (-12.0, 49.0) |  | 2283.2  (2006.7, 2561.8) | 103.2  (90.3, 117.2) |  | 2701.81  (2246.5, 3189.1) | -0.1  (-20.4, 25.0) |
|  | Total | 276.7  (241.3, 322.0) | 86.0  (73.2, 99.0) |  | 153.91  (132.6, 176.7) | 32.3  (8.6, 59.0) |  | 2022.0  (1799.8, 2264.8) | 106.0  (93.7, 118.8) |  | 3230.5  (2799.1, 3679.5) | 13.7  (-5.3, 36.1) |
| Age-standardized rate,  per 100 000 | Male | 209.8  (185.5, 239.4) | -8.0  (-14.3, -1.7) |  | 170.3  (141.9, 200.2) | -30.8  (-45.8, -13.4) |  | 1325.4  (1181.0, 1486.0) | 16.3  (9.8, 23.0) |  | 3052.1  (2535.2, 3643.0) | -34.5  (-49.0, -16.5) |
|  | Female | 194.5  (169.7, 225.2) | -10.1  (-16.3, -3.5) |  | 97.4  (78.9, 117.0) | -48.2  (-60.2, -33.1) |  | 1583.5  (1403.7, 1771.4) | 11.4  (5.4, 18.2) |  | 1876.8  (1566.5, 2204.5) | -49.4  (-59.6, -36.3) |
|  | Total | 200.8  (177.0, 230.8) | -9.3  (-15.5, -3.3) |  | 127.3  (110.2, 144.9) | -39.8  (-50.7, -28.6) |  | 1468.9  (1309.2, 1640.1) | 13.2  (7.7, 19.1) |  | 2412.5  (2102.9, 2742.5) | -41.6  (-50.9, -30.7) |

# Supplementary Figures





**Supplementary Figure 1.** Incidence, prevalence, deaths and DALYs rates of ischemic stroke, intracerebral hemorrhage, and subarachnoid hemorrhage in different age groups in China in 2019

**Supplementary Figure 2.** Attributable DALYs (95% UI, in thousands) by stroke risk factors for all ages in China, 2019. *Air pollution cluster includes ambient particulate matter pollution and household air pollution from solid fuels. Non-optimal temperature cluster includes low ambient temperature and high ambient temperature. The tobacco cluster includes smoking and second-hand smoking. Dietary risks cluster includes a diet high in red meat, a diet high in sodium, a diet low in fibre, a diet low in fruits, a diet low in vegetables, a diet low in whole grains, and alcohol use. Behavioural risks cluster includes tobacco cluster, dietary risks cluster, and low physical activity. Environmental risks cluster includes air pollution cluster, non-optimal temperature cluster, and lead exposure. Metabolic risks cluster includes high fasting plasma glucose, high LDL cholesterol, high systolic blood pressure, high body-mass index, and impaired kidney function.
